# Role of Knowledge Management in Development and Lifecycle Management of Biopharmaceuticals

**DOI:** 10.1007/s11095-016-2043-9

**Published:** 2016-10-26

**Authors:** Anurag S. Rathore, Oscar Fabián Garcia-Aponte, Aydin Golabgir, Bibiana Margarita Vallejo-Diaz, Christoph Herwig

**Affiliations:** 1 Department of Chemical Engineering, Indian Institute of Technology Delhi, Hauz Khas, New Delhi, India; 2 Research Area Biochemical Engineering, Institute of Chemical Engineering, Vienna University of Technology, Vienna, Austria; 3 Department of Industrial Engineering, Universidad Nacional de Colombia, Bogotá, Colombia; 4 Department of Pharmacy, Universidad Nacional de Colombia, Bogotá, Colombia; 5 CD Laboratory on Mechanistic and Physiological Methods for Improved Bioprocesses, Vienna University of Technology, Vienna, Austria

**Keywords:** knowledge indicators, knowledge management, ontologies, process modeling, quality by design

## Abstract

Knowledge Management (KM) is a key enabler for achieving quality in a lifecycle approach for production of biopharmaceuticals. Due to the important role that it plays towards successful implementation of Quality by Design (QbD), an analysis of KM solutions is needed. This work provides a comprehensive review of the interface between KM and QbD-driven biopharmaceutical production systems as perceived by academic as well as industrial viewpoints. A comprehensive set of 356 publications addressing the applications of KM tools to QbD-related tasks were screened and a query to gather industrial inputs from 17 major biopharmaceutical organizations was performed. Three KM tool classes were identified as having high relevance for biopharmaceutical production systems and have been further explored: knowledge indicators, ontologies, and process modeling. A proposed categorization of 16 distinct KM tool classes allowed for the identification of holistic technologies supporting QbD. In addition, the classification allowed for addressing the disparity between industrial and academic expectations regarding the application of KM methodologies. This is a first of a kind attempt and thus we think that this paper would be of considerable interest to those in academia and industry that are engaged in accelerating development and commercialization of biopharmaceuticals.

**Electronic supplementary material:**

The online version of this article (doi:10.1007/s11095-016-2043-9) contains supplementary material, which is available to authorized users.

## INTRODUCTION

### Relevance of Knowledge Management for Pharmaceutical Quality Systems

**Fig. 1:**
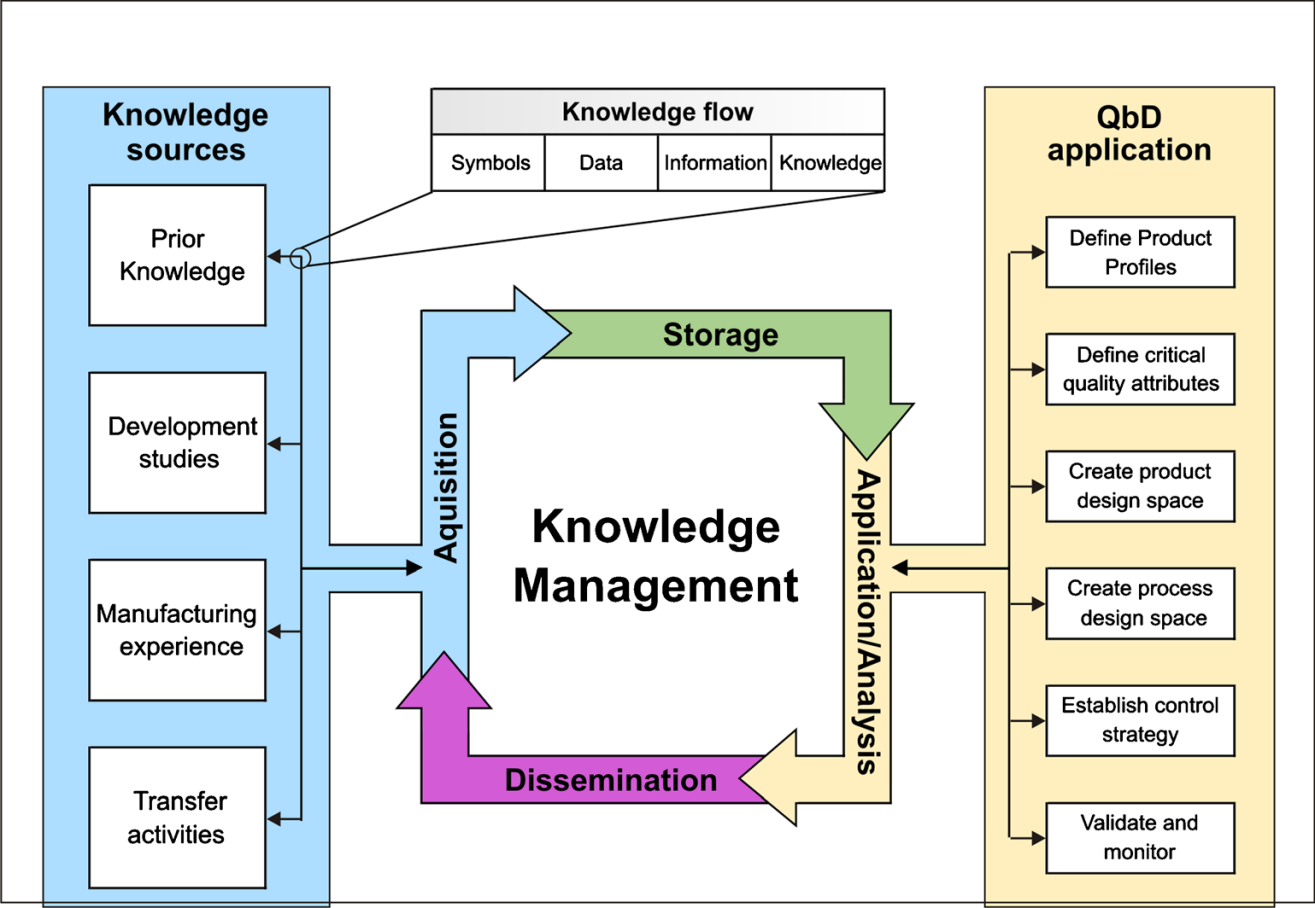
Illustration showing the link between the various sources of knowledge and QbD applications by knowledge management functions. Knowledge flow starts from the four possible sources and may consist of symbols (numbers, letters), data (collections of symbols), information (data in context), and knowledge (applied information). KM uses four distinctive functions to efficiently guide the flow of knowledge towards achieving QbD goals as required to gain product and process understanding.

Quality by Design (QbD) has been defined in the ICH Q8 guideline as “a systematic approach to development that begins with predefined objectives and emphasizes product and process understanding and process control, based on sound science and quality risk management” (1,2). This is a science and risk based approach of product development and includes steps of identification of the product attributes that are of significant importance to the product’s safety and/or efficacy (Quality Target Product Profile and Critical Quality Attributes); design of the process to deliver these attributes; a robust control strategy to ensure consistent process performance; validation and filing of the process demonstrating the effectiveness of the control strategy; and finally ongoing monitoring to ensure robust process performance over the life cycle of the product (Fig. 1).

Knowledge management can play a key role in facilitating efficient transfer and utilization of information to facilitate the above mentioned tasks. For example, a major task in implementing Quality by Design (QbD) is to holistically review knowledge coming from a disparate variety of sources and then use it to assess criticality of the various quality attributes (1,2). It is unlikely that the manufacturer has specific information about each and every attribute for a given biotherapeutic to assess its criticality. Most likely, the manufacturer will be making a decision based on what has been reported in literature and prior experience with other similar product. Further, QbD necessitates that knowledge gained over a product’s lifecycle can be utilized in an environment of continuous improvement (1,2). Data generated during a life-cycle can be used to start QbD for the next product and/or create an ‘improved’ product. With the widespread adoption of QbD principles by the biopharmaceutical industry, and the growing emphasis on enhanced process understanding, the need for establishing efficient KM strategies is more than ever (3). KM can be quite effective in managing the flow of information towards development of design space, control strategies, and technology transfer as well as use this information towards continuous improvement of the process and the product (4).

Within this context, the International Conference on Harmonization (ICH) defines Knowledge Management (KM) as “a systematic approach to acquiring, analyzing, storing and disseminating information related to products, manufacturing processes and components” (5). In the field of biotechnology, this definition can be interpreted as consisting of capturing, structuring, retaining and reusing information in order to understand a particular biological system and to deliver the gained knowledge to an information system (6,7). The information system should ideally be able to capture the relevant knowledge, deliver it to the relevant user, and invest it to improve organizational and/or individual performance (8).

### Classification of Knowledge Management Tools

To clarify the prevalence of different KM tools in the academic literature and their perceived industrial relevance, a classification of KM tools based on functionality is needed. KM tools can be arranged inside the four basic dimensions proposed in the ICH guidelines: acquisition, analysis, storage and dissemination (Table I), similar to previous efforts (41–43). The ICH Q8 guideline makes reference to the need for KM in the form of “The information and knowledge gained from pharmaceutical development studies and manufacturing experience provide scientific understanding to support the establishment of the design space, specifications, and manufacturing controls” and “Changes in formulation and manufacturing processes during development and lifecycle management should be looked upon as opportunities to gain additional knowledge and further support establishment of the design space” (44). Similarly, ICH Q9 discusses the role of knowledge in assessing risk as well as in activities such as change control (45). In an earlier publication, we identified a set of 12 KM tools commonly applied by pharmaceutical organizations throughout their products’ lifecycle (7). Since it is necessary to involve strategy, organizational culture, and cooperation between departments in any KM system (46), in this work, we expanded the classification by an additional supporting dimension consisting of four tools: KM models, strategies, indicators, and frameworks.

**Table I:** Functional Classification of KM Tools. Adapted from (7)

*Class*	Definition	References
Acquisition		
*Knowledge and data capture*	Allows gathering and integration of data, going from basic paper to software and hardware applications with functionalities similar to a scientific notebook. It includes electronic laboratory notebooks, data logging and best practice reports.	Data integration platforms for R&D production (9), electronic data capture supporting lab work (10).
*Retrieval*	Tools used to obtain specific data from structured sources through use of Query and Answer platforms or an automated intelligent agent.	Medical information retrieval (11), retrieval in dynamic data management for chemical process operations (12)
*Data and text mining*	Applications that explore large volumes of data to find hidden patterns, creating previously unknown information from it.	Mining biological molecules structure (13), visual data mining (14).
Storage		
*Knowledge and data bases*	Secondary sources for retrieval at different levels of abstraction including those with very complex forms of knowledge. They have several dimensions to be catalogued in, like syntactic vs. semantic integration, warehousing vs. federation, declarative vs. procedural access, generic vs. hard coded and relational vs. non-relational based data model.	Databases in regulatory reviews (15), libraries for clinical trials (16), data repositories for chemical kinetics (17).
*Maps, Taxonomies and ontologies*	While taxonomies and maps are hierarchical representations of knowledge, ontology defines and semantically describes the data and information, being the basis for modeling different forms of knowledge. They create a common, explicit, and platform-independent vocabulary that is both machine accessible and human usable.	Holistic ontologies in pharmaceutical engineering (18), ontologies for pharmaceutical mathematical knowledge modeling (19)
Analysis		
*Visualization*	Information arrangement that displays a set of data easily understood by a wide audience. Graphs, charts and Response Surface Methods are included.	Visualizing metabolic networks (20), Response Surface Methodology in biotechnology (21)
*Statistical analyzers*	Provide quantitative measure of the inherent variability of a phenomena, assessing the current state of control and enabling process improvement.	Chemometrics-based PAT (22), Integrated statistical platforms to laboratory management (23)
*Intelligent agents*	Decision support tools that are grounded in the emulation of skills for reasoning and inference. Their system is composed by a knowledge base, an inference engine and an interface for the user.	Intelligent process management in continuous pharmaceutical operations (24), intelligent decision support in pharmaceutical development (25)
*Models and simulation*	Effective integration of knowledge, information, and assumptions with experimental data in one unified representation, to predict outcomes and express relationships.	Modeling in decision making for drug development (26), multiscale pharmaceutical mechanistic modeling (27)
Dissemination		
*Network and web technologies*	Interfaces and platforms that help to interconnect resources inside the boundaries of an organization, exploiting the knowledge within the firm. Externalization can be achieved through the use of web technologies (e.g. corporate portals) aiming to the establishment of virtual organizations.	Pharmaceutical factory monitoring system based on Ethernet (28), virtual organizations for pharmaceutical engineering and science (29)
*Collaboration*	Community participation applications like groupware, collaborative project management software and electronic conferencing tools, used for accessing to a firm’s external knowledge or facilitating research partnerships.	Communities and collaboration in discovery and development (30), collaborative drug discovery (31)
*Document management systems*	This tools are found in the interface between storage and dissemination, they locate documents inside a clear management police and keep track of their status and versions.	Modern document management in the regulatory context (32), electronic document management (33)
Support		
*KM frameworks*	An organized set of ideas, principles, information, rules and definitions that configure the structure of a knowledge management initiative.	Frameworks in process KM (4), a framework for knowledge-based diagnostic systems in batch chemical processes (34)
*KM models*	A theoretical representation of the desired or actual state of a system, making special emphasis on the knowledge actors, flows, constrains and relationships	Process model for knowledge management in plant maintenance (35), structural model of knowledge management across borders (36)
*KM strategies*	Plan or direction to achieve the goals of knowledge management so it can react to uncertain environments.	Strategies for drug knowledge transfer process in pharmaceutical marketing (37), strategies to manage knowledge flows between high-tech firms and universities (38)
*KM indicators*	Figures that represent the level of success for a given knowledge management activity, aiming to assess its performance in concordance with a stated goal in the KM strategy	Measurement scale for knowledge management in biotechnology (39), Structural influence index in KM (40)

### Analyzing Knowledge Within Pharmaceutical Systems

The use of KM tools in biopharmaceutical production systems can further be organized by the type of knowledge being analyzed and the specific QbD activities addressed. According to the relevant ICH guidelines (Q8–Q11) and the forthcoming Q12 guideline about lifecycle management, several knowledge sources typically need to be accounted for, including prior knowledge, pharmaceutical development studies, technology transfer activities and manufacturing experience (5,44,45,47,48). Therefore, each application of KM tools can be based on these four different classes of knowledge sources as shown in Fig. 1. In addition, it is necessary to have the final destination of the knowledge flow in mind. The use of each KM tool can be studied based on the specific QbD activities addressed, primordially: design space establishment, risk management, development of control strategies, and identification of CQAs. In addition, since many literature sources do not directly link the application of tools to specific QbD issues, general categories that are frequently used to describe KM benefits, such as manufacturing facilitation, enhancement of body of knowledge, regulatory flexibility, and innovation support have been introduced.

## METHODOLOGY

To gain insights about the status of various KM tools within the academic literature, an exhaustive non-systematic literature review was conducted according to a defined protocol (available in the Supplementary Information section). The following databases were searched: ScienceDirect (search date: 02.11.14), Embase (search date: 02.11.14), IEEE (search date: 02.11.14), Scopus (search date: 06.11.14), Springer (search date: 20.11.14) and Taylor & Francis (search date: 02.11.14). All publications that contained the term “Knowledge Management” in combination with the words “pharmaceutical,” “biotechnological,” “drugs” or “chemical” were selected.

With this search strategy, a total of 5203 records were found after removing duplicates, without employing any other kind of information source or contacting authors for further information. A manual selection was performed to assure that the studies were fitting into the framework for pharmaceutical development and quality systems within the QbD approach. This was done in the first instance by reading each of the paper’s titles and discarding all of those that did not fit into the pharmaceutical, chemical or biotechnological sectors or that did not correspond to the title of a research paper; in some cases, this screening was supported with the analysis of the paper’s abstracts. In the second instance, those records that through the analysis of their abstracts could not be fitted within the scope of the pharmaceutical product lifecycle, as defined by the ICH guidelines, were also discarded. A detailed description of the used inclusion and exclusion criteria can be found in the review protocol (Supplementary Information). Based on this process, the final literature assessment resulted in 356 publications.

The data was extracted from the records manually via the evaluation of the full text, when it was available. The selected publications described 503 application scenarios involving a wide spectrum of KM tools/solutions for solving pharmaceutical and biotechnological issues. A classification of each KM tool was performed according to the type of the employed tool (Table I), managed knowledge source, and the intended QbD-related objective (as presented in Fig. 1).

To compare the results of this literature review with the actual perceptions within the biopharmaceutical industry, a consultation with employees at R&D, management, process development and regulatory affairs (*n* = 34) from 17 major biopharmaceutical organizations was performed *via* an online questionnaire. The participants were asked to rank the importance of the different KM tool classes with respect to application towards various QbD-related knowledge sources, the KM support tools were not included in this questionnaire, analyzing just the process perspective addressed by the ICH in its definition for KM. Next, we focused in the four main functions: acquire, store, use, and distribute knowledge. In addition, the respondents ranked the applicability of various QbD-related knowledge sources towards achievement of specific QbD objectives. According to the area of most expertise (QbD-related knowledge sources), each participant was asked to rank the extent of application of the 12 tool classes in their organization. The following scoring system was used: a score of (2) for being used, (1) for not being used but considered useful, (−1) for not considered useful, and (0) for not knowing the tool. The quantitative results from the literature survey were normalized versus the actual frequency of the four KM functions, and used for comparison with the results found from the industrial questionnaire.

The ethics committee/institutional review board was not involved in the approval of the study, because no personal information was collected. The participants were informed in writing of the scientific purpose and objectives of the study. In addition, they were asked to consent to the participation. The participants provided their written consent to participate in this study. The participant consent was recorded in the survey form. In addition, without the consent, the participant was not allowed to proceed in the survey. No effort was made to anonymize IP addresses as this information was not visible to researchers.

Based on the outcome of this analysis, three issues were detected as having a high priority and were explored further: 1) Knowledge indicators, as the least developed KM tool 2) Taxonomies and ontologies as the most used KM tool in the literature and 3) Modeling and simulation, due to its perceived importance for the creation of design space and potential for storage of QbD-related product and processes knowledge.

## REUNITING THE VIEWPOINTS: KM IN THE INDUSTRY AND ACADEMIA

### Assessment of KM in the Published Literature

**Fig. 2:**
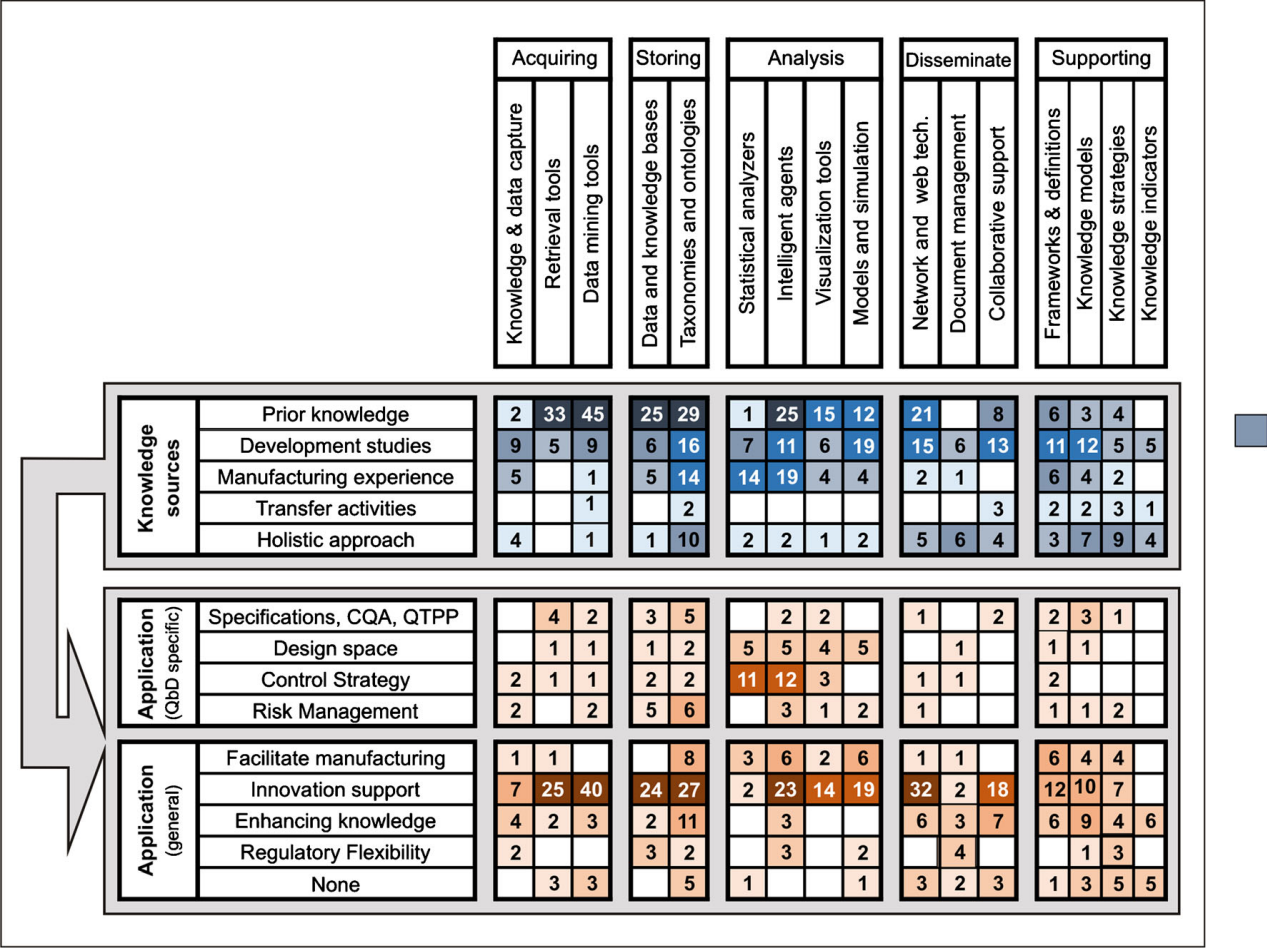
Distribution of KM applications in the published literature used in various sources of knowledge, as well as in QbD-specific applications. Each number in the matrix indicates the total number of KM tools found in the scientific literature that fall within the corresponding categorization. The columns of the matrix indicate the type of KM tools. The first 5 rows (Knowledge sources) show the distribution of KM tools that address knowledge stemming from specific sources of knowledge as specified by the ICH guidelines for QbD approaches. The lower rows mention the applications of knowledge management tools to specific and general QbD objectives. Hence, as illustrated in Fig. 1, the knowledge sources (upper rows) are linked by knowledge management tools (columns) to QbD applications (lower rows).

**Fig. 3:**
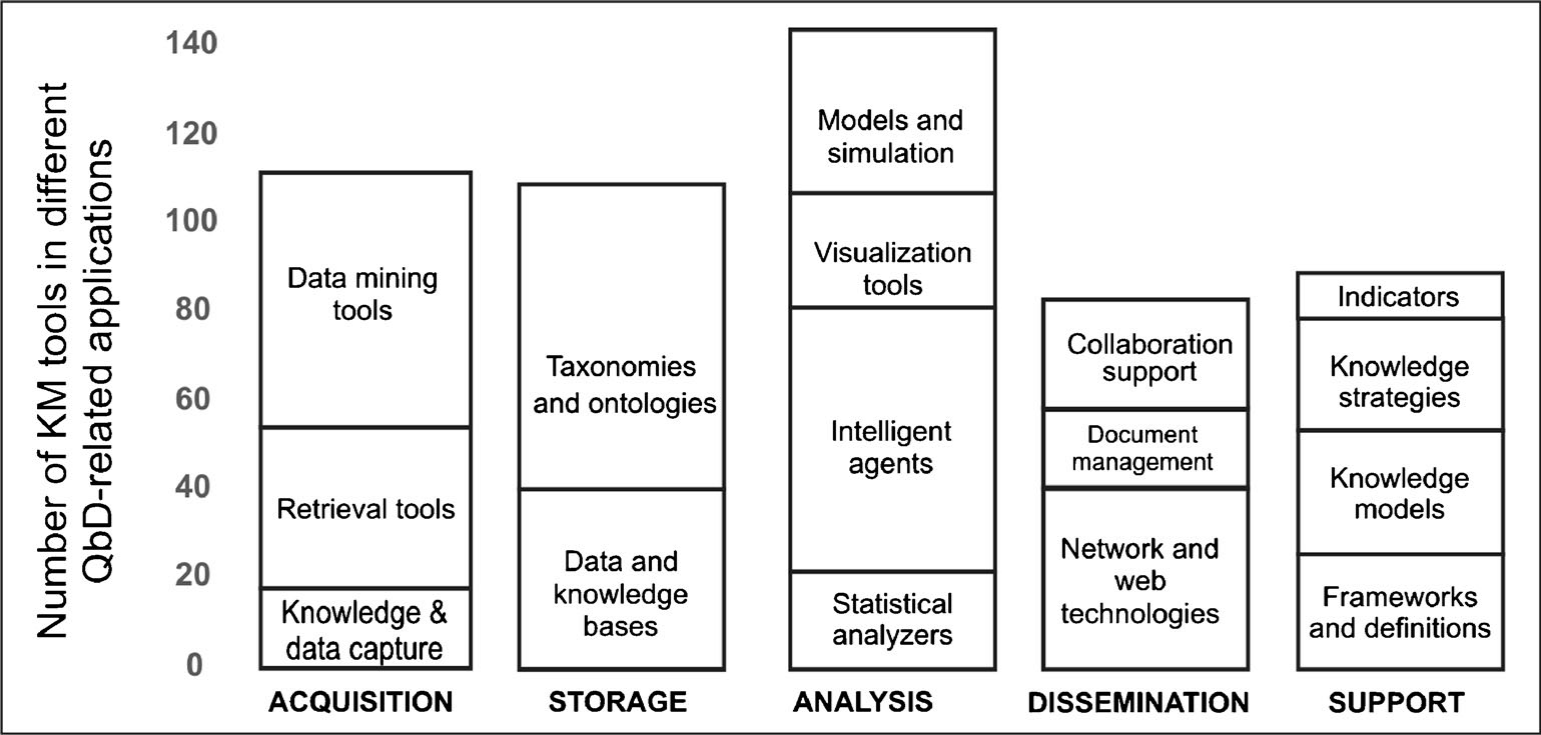
Number and type of KM tools for solving specific QbD-related applications. KM tools based on data mining, taxonomies and intelligent agents are the most used tools and are mainly based on data driven algorithms. Mechanistic approaches such as model and simulations have an increasing trend but are less used so far.

Several authors have reported applications of KM principles towards a wide range of pharmaceutical-related tasks. Published literature included paper that have appeared in international peer reviewed publications. Categorizing each article in the review pool according to the previously discussed criteria in terms of KM tool group, knowledge source, and QbD-related objectives, allowed the analysis of the state of the art from the perspective of a life-cycle paradigm. A visualization of the use of the different KM toolsets within the reviewed academic publications with respect to the type of knowledge being managed, and the intended QbD-related objectives is provided in Fig. 2. The matrix reveals the results from the bibliographic research, categorizing the type of the tool (columns), the type of the knowledge source (blue rows), and the intended applications (red rows). The four major Knowledge sources were identified from the analysis of the ICH’s Quality Guidelines, being condensed into: Prior knowledge (biological, chemical, and engineering principles, literature, experience and information from the development of similar drug products), pharmaceutical development and innovation (information from the use of a scientific approach to understand a product in its development phase), technology transfer activities (gained through the transference of product and process knowledge from development to manufacture and from manufacturing sites), and manufacturing experience (day-to-day knowledge from routine commercial manufacturing of the drug product). The final outcome is colored according to the number of publications, which allows the identification of over- and under-represented areas. A comparison of the number of KM tools for solving each of the QbD-related applications is provided in Fig. 3. In our review, most of the tools were classified in the “Analysis” category. The categories for knowledge acquisition and storage also contained a large number of reported tools.

As shown in Fig. 2, some of the reviewed technological solutions are found to be concentrated on using specific sources of knowledge. For instance, there is a predominance of statistical analysis tools that use knowledge from manufacturing experience, such as multivariate data analysis of spectroscopic datasets (22,49), chemometric tools for implementation of process analytical technologies (PAT) (23), and Intelligent Agents that supervise biotechnological processes (50)). Aside from their major role in manufacturing, these statistical analysis tools are also used for the management of development information (51). Databases, visualization, retrieval, and data mining tools are focused on prior knowledge due to their role in the maintenance of knowledge bases as a core element of a lifecycle-oriented quality systems. Data capture, collaboration, and document management tools are linked predominantly to knowledge from development activities. They perform tasks required for novel data generation and storage, as it is critical to codify new datasets making them traceable and available to further lifecycle stages. KM models have been used as tools that guide the flow of information present in research and development activities. For instance, they have been used for creating innovation through KM in the pharmaceutical industry (52,53), for relating intellectual capital and organizational performance through intermediate variables, enablers, and learning flows (54), for presenting technical details of a workflow-based KM system for manufacturing of liquid-based drug products (55), for formulating decision analysis frameworks for risk management (56), and even for developing a KM model to transfer drug related information in pharmaceutical marketing (37). Such diverse applications of KM models demonstrate their important role within the lifecycle of a pharmaceutical product.

Furthermore, there are some tools that are applied holistically, not dealing with a single source of knowledge but with a combination of them. For instance, applications of taxonomies and ontologies, intelligent agents, network and web technologies and models and simulations can be found across a wide spectrum of knowledge sources. These technologies will be discussed in more detail in “Indicators: There is no Management Without Metrics”, “Ontologies: There is no Management Without Order” and “Role of Modeling in Facilitating KM” sections. Regarding tools that support KM, since KM frameworks and strategies constitute the basis for a structured KM policy in any organization, a unified consensus and a standardized set of definitions and guidelines for the industry is needed. Currently, the proposed frameworks have little in common and address the same issues with an uneven perspective and great dispersion.

Compared to other sources, knowledge from technology transfer activities (academia to industry or within industry) is covered less frequently by the reviewed articles. As it will be shown in later sections, the results of a survey among industrial representatives revealed that information related to transfer activities is often considered as sensitive and hence is not disclosed in the public domain. Also, transfer and validation activities are located in the interface between development and manufacturing, and it is often difficult to address this issue in purely academic settings (7).

Looking at the target of KM activities, enhancement of innovation is found to be a major goal pursued in the investigated publications. Increasing the body of knowledge is the second non-specific QbD goal targeted by several authors. Regulatory flexibility has also been explored through the study of regulatory information management systems (57), modeling for pharmacometrics (58), application of document management systems (33), use of databases, libraries and reporting templates (15), and use of information platforms (59).

Use of knowledge and data capture tools is underestimated in the published literature for the establishment of CQAs and Design Spaces. While models and simulations are quite commonly used for the establishment of control strategies in the industry, most of these applications are not disseminated publicly and only a few such examples could be located such as the improvement of manufacturing processes through the modeling of chemical plants (60) and research and development through integrative multi-scale modeling frameworks (27).

### An Industrial Perspective of KM

**Fig. 4:**
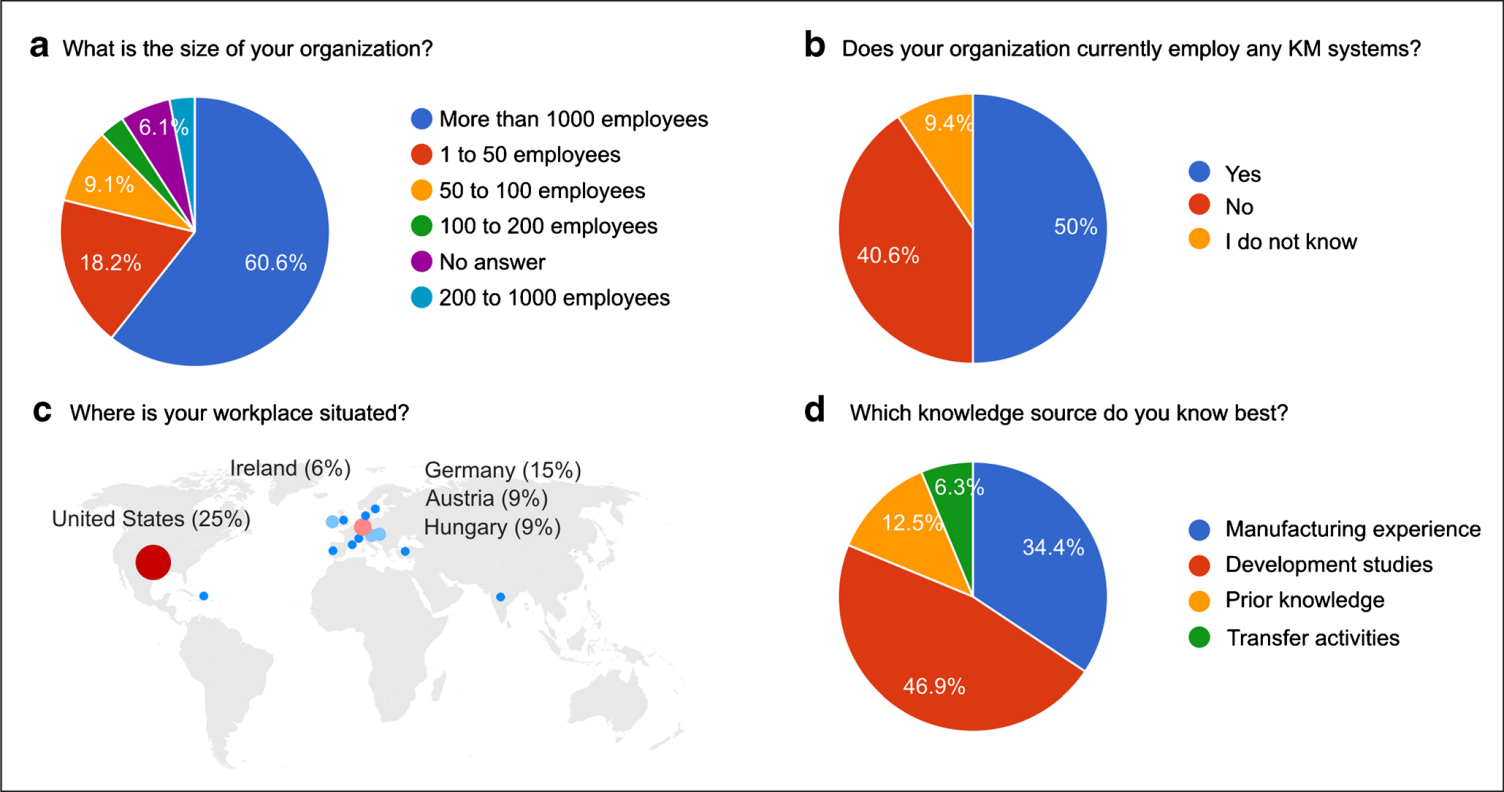
Results of an online survey conducted among biotechnology professionals. (**a**) Size of affiliated organizations, the majority is large organizations, but also SMEs. (**b**) Existence of KM systems at the corresponding organizations. Only half of the organizations, thereof mainly large organizations, used KM systems. (**c**) Geographical location, mainly industrial countries were included in the survey (**d**) Professional experience with different sources of knowledge, classical fields of process and manufacturing science are supported by KM tools, while QbD related tasks (e.g. prior knowledge) are less addressed.

For analyzing perceptions within the biopharmaceutical industry, consultation with employees (*n* = 34) of 17 major biopharmaceutical organizations was performed *via* an online questionnaire. As shown in Fig. 4a, most respondents belonged to organizations with more than 1000 employees, and almost half of the respondents indicated to have some kind of KM system in place in their respective organizations (Fig. 4b). The majority of respondents indicated to be based in either the United States or within Europe (Fig. 4c). Responding to the question of “which knowledge source do you have the most experience with?”, 46.9% indicated to be most familiar with knowledge from “development studies,” followed by 34.4% who indicated to be more familiar with knowledge from “manufacturing experience” (Fig. 4d).

**Fig. 5:**
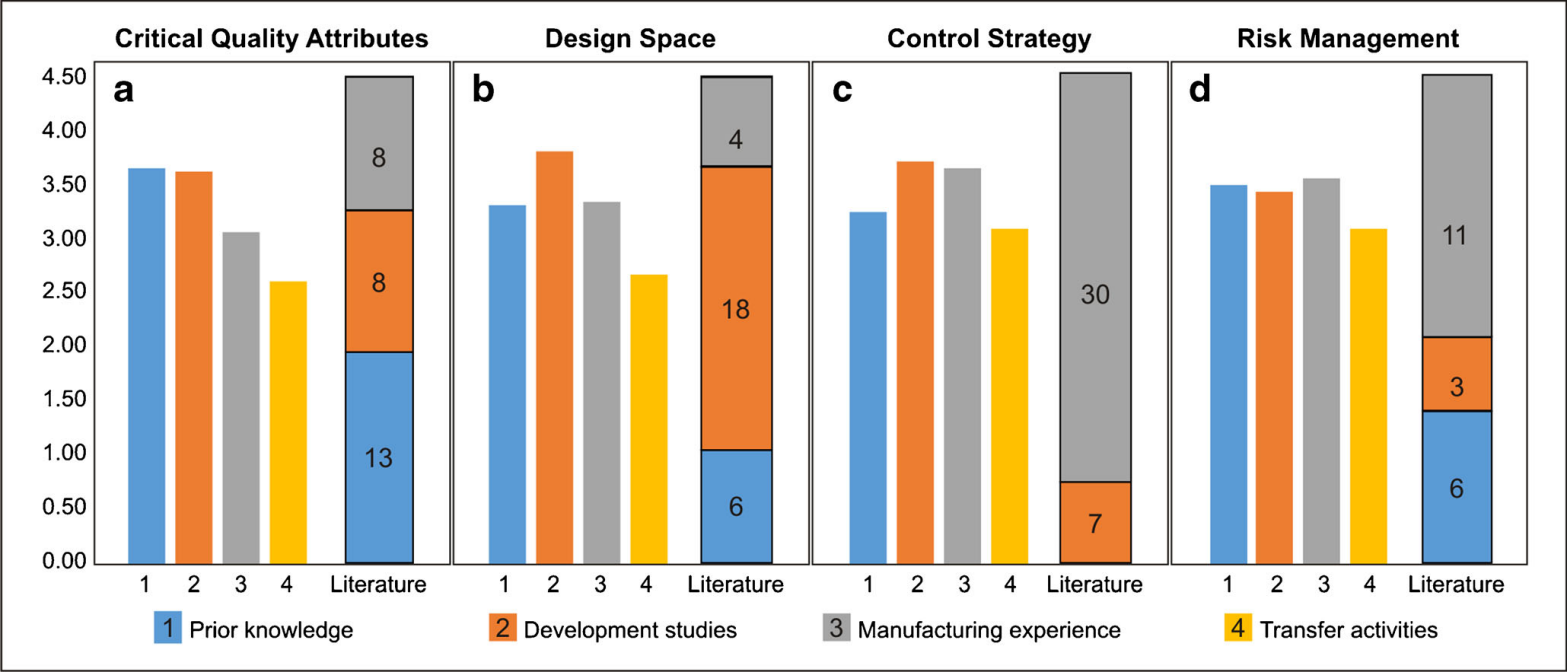
Importance of the knowledge sources for achievement of QbD goals rated by professionals vs. the number of tools in the literature. Comparison of the importance rating given by the industrial respondents to each of the knowledge sources in the achievement of the main QbD goals to literature, represented in bar plots ranking from 0 to 4.5. Aside each set of bars, a segmented plot shows the frequency found in the literature for specific KM tools used in the same four main QbD goals; the color key corresponds to the same classification of sources applied in the bar plots.

Figure 5 let us to compare the importance rating given by the industrial respondents to each of the knowledge sources in the achievement of the main QbD goals; this point of view is represented in bar plots ranking from 0 to 4.5. Aside each set of bars, a segmented plot let us see the frequency found in the literature for specific KM tools used in the same 4 main QbD goals; the color key corresponds to the same classification of sources applied in the bar plots. As it was expected, there are no tools in the literature that manage transfer activities’ knowledge to support the achievement of the QbD goals and there are no tools using prior knowledge for the establishment of control strategies. For the definition of the CQAs, if we follow the importance given by the industry, we should expect a similar frequency of tools using prior and development knowledge. Instead there is a deficit in development of such tools. Design space establishment is dominated by tools that manage knowledge from development, with only a few targeting new applications for managing information from manufacturing. Finally, there is a misbalance in the tools targeting development of control strategy: while the industrials give a similar importance score for both the development and manufacturing knowledge in the achievement of this goal, the actual frequency in the literature prioritizes manufacturing knowledge over the development knowledge by a nearly 1:4 ratio.

### Comparison of Academic and Industrial Perspectives

**Fig. 6:**
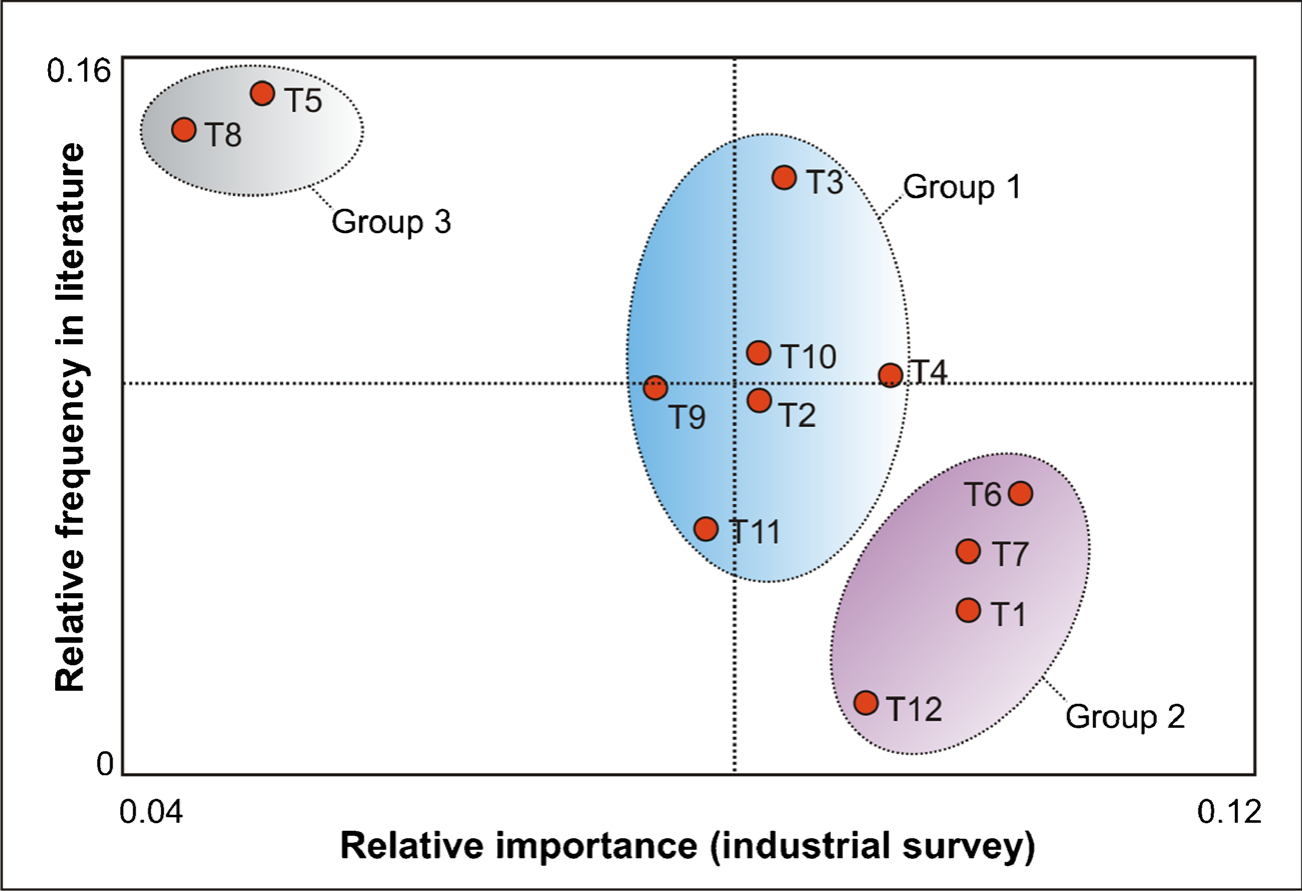
Comparison between the relative frequency of a tool reported in the literature and the relative importance rating addressed by the industrial questionnaire. Group 1 represents the technologies which are recognized as being modestly important by the respondents of the survey, and also receive a significant amount of attention from the academic community. The technologies included in Group 2 are ranked as relevant by the industry but not congruently represented in the literature. The technologies included in Group 3 are perceived as less important by the industry yet addressed highly by the scientific community. Mean values are shown as dotted lines. T1: Capture tools, T2: Retrieval tools, T3: Data mining, T4: Data and knowledge bases, T5: Taxonomies and ontologies, T6: Visualization tools, T7: Statistical analyzers, T8: Intelligent agents, T9: Models and simulation, T10: Network and web technologies, T11: Collaboration tools, and T12: Document management systems.

A comparison of the reviewed literature and the results obtained from the industrial questionnaire was performed, addressing the relative frequency of the KM tools in the literature (the ratio of a tool’s usage compared with the use of all kinds of KM tools), and the relative importance rating defined by the industry (averaging the scores given when the participants were asked to rate the importance and use of a tool in their organizations). This exercise allowed us to identify three distinct groups of KM tools, as shown in Fig. 6. The first group represent the technologies which are recognized as being modestly important by the respondents of the survey (centered on the mean), and also receive a significant amount of attention from the academic community. These include technologies, such as knowledge retrieval tools (T2), network and web technologies (T10), databases (T4), and data mining tools (T3). The technologies included in Group 2 are ranked as relevant by the industry but not congruently represented in the literature (e.g. visualization tools, statistical analyzers, capture tools, and document management systems). On the other hand, the tools included in Group 3 were found to be commonly addressed by literature sources and yet not ranked as important by industrial representatives (ontologies and intelligent agents).

Group 2 is composed of tools which are widespread and traditionally accepted for their knowledge management attributes. The reason for the lack of attention by the scientific community is perhaps a lack of interest as a result of their perceived limited value towards research. However, these primary tools continue to be relevant, as there is an industrial need for rethinking their functionalities in the QbD paradigm. For instance, data flow automation as an enhanced knowledge capture tool, diminishes manual documentation and the related control activities, boosting the efficiency up to a 30% for laboratory staff and management (10). A shift towards extraction of know-how and tacit knowledge (61) as opposed to the classic explicit data capture approaches also represent new direction for research in this field. Similarly, visualization tools have recently been enhanced for visual data mining (14) and interactive hypothesis generation (62). These tools have also been combined with other KM tools as in the case of ontology-related semantic graphs for hypothesis generation (63). The challenge for these classic tools is to be continuously reinvented according to the demands of data-rich environments and the complexity of their multivariate relationships.

The tools included in Group 3, perceived as less important by the industry yet addressed highly by the scientific community (consisting of taxonomies and intelligent agents) can be considered as technologically more demanding. These are clearly areas for further research to realize potential benefits that will result from their industrial implementation. However, the reason for the discrepancy between the rankings is likely due to a lack of thorough understanding by the industrial respondents of the tools, perhaps as a result of their higher complexity. Additionally, a contributing factor might be a gap in the understanding of industrial needs by academic researchers.

From this analysis, we highlight three tool classes that are highly valuable for further discussion within the pharmaceutical community. These will be discussed together with few examples in the later sections.

1. KM indicators appear to be the least developed resource and have not been applied to any QbD specific goal, despite being praised in management areas for give direction to different kind of initiatives (64,65).
2. Taxonomies and ontologies, which are often linked with artificial intelligence, are recognized by the academic community as powerful tools for managing knowledge within the pharmaceutical research field.
3. Simulation and modeling have been explored to a larger extent due to their connection with QbD implementation: product and process understanding. Modeling fulfills the definition of a functional design space, representing an efficient knowledge summary, which is able to explain the behavior of a given system on a mechanistic level.

## PROMISING KM TOOLS FOR PHARMACEUTICAL PRODUCTION SYSTEMS

### Indicators: There is no Management Without Metrics

An indicator is a quantitative or qualitative measure which provides information on the status of a system and is a reference for supporting appropriate decision making. They are grounded in the measurement of performance figures detected as markers of success or failure for a definite activity or set of activities. Therefore, delineation of factors that impact an activity’s success is a critical step for the establishment of indicators. In this direction, a previous work outlined 32 variables for pharmaceutical KM systems, underlining seven critical factors: benchmarking and knowledge structure, organizational culture, information technology, employee involvement and training, leadership and commitment of senior management, learning environment and resource control, and professional training evaluation (46). In the same field, without focusing on the pharmaceutical industry, the inclusion of knowledge practices in strategic planning has also been analyzed (66). The authors weighed product leadership, customer intimacy, and operational excellence, to discover a direct relation between knowledge and intermediate organizational performance metrics.

Once the link to a certain performance measurement is established, clearer indicators can be generated. An example of this can be found in the study of individual performance through the assessment of attributes and actions. Ten individual knowledge indicators have been proposed, composed by knowledge stock indicators (education, training, experience, IT literacy), knowledge flow indicators (business communications, business process interactions, personal network), knowledge utilization indicators (performance and creativity), and one indicator related to financial figures (67).

Another approach has been proposed through the use of patent-based performance metrics to reveal the scientific and technological components of a pharmaceutical firm’s knowledge base (considered as its ‘core’ element). The patent’s metrics have been correlated with four innovative performance variables: the scope of firm knowledge base, research expenditures, firm size, and external knowledge flows (68). This can be complemented with the bigger task of evaluation of the intellectual capital as a whole. A recent study in this field accounted for human capital (learning and education, experience and expertise and innovation and creation), structural capital (systems and programs, research and development and intellectual property rights), and relational capital (strategic alliances, licensing and agreements and relation and knowledge about partners, suppliers and customers). The authors proposed 17 human, 11 structural and 14 relational capital indicators (69). These performance-based indicators have some complications, as measurement of an entire organization’s KM performance is difficult to assess from the perspective of process, leadership, culture or technology. Better efficiency and effectiveness in KM performance can be achieved through a project-orientated approach (70). The latter is relatively unexplored in the pharmaceutical field.

A firm’s knowledge strategy can also be addressed in three dimensions: the emphasis on speed of learning (technology cycle length), emphasis on internal sourcing (R&D spending) *versus* external sourcing of knowledge (R&D activities closeness to basic science), and the development of a broad *versus* a narrow knowledge base (through the patent’s dispersion index) (71). Furthermore, a recent survey has examined how biotechnology companies placed value on knowledge sources that supported R&D, including elements such as the existence of scientific databases, the policies to protect IP, employee education, and information derived from industry and public sources (72). Top priority was given to the IP protection, followed by the firm’s scientific database and employee education was ranked third of the knowledge assets.

### Ontologies: There is no Management Without Order

Chaos cannot be managed, and knowledge is easily accessed when it is transferable to information systems. Because of this, a common, explicit, and platform-independent vocabulary that is both machine accessible and human usable is needed to streamline the generation and flow of knowledge (18). An ontology defines and semantically describes data and information. This serves as the basis for modeling different kinds of knowledge, avoiding difficulties around the organization of related information and the lack of open and systematic ways to manage meta-data (25). Taxonomies (hierarchies of data in classes) and ontologies (explicit description of classes by their properties) can thus serve as the spinal cord of the KM systems over which further knowledge applications like intelligent support engines and databases can be developed.

As a core in many knowledge-based systems, taxonomies and ontologies exhibit several characteristics needed for the four functions of KM to be integrated, leading to the unification of vocabularies towards the fulfillment of lifecycle initiatives. Therefore, several authors have extensively studied them in different areas: risk management (73,74), product formulation, unit operation model integration (18), determination of CQAs and CPPs (75), mathematical model storage, use, and solving (19), regulatory compliance (76), manufacturing execution systems (77) and drug discovery (78).

Ontologies are also fundamental in intelligent reasoning systems, which usually possess three components: a knowledge base where information from a specific process is stored continuously, an inference engine that makes the reasoning possible, and an interface that translates the findings to a common language for the user. The inference engine has a logical component that generally has an ontological structure and can vary in complexity, coming from single agents to modular systems where agents interact with one another to achieve individual objectives by exchanging information, cooperating or negotiating to solve conflicts (79).

### Role of Modeling in Facilitating KM

Growing manufacturing costs and increased demands for reducing the time and resources required for development of production processes have led to an increased interest towards model-based approaches for optimization of biochemical processes. From the viewpoint of KM, process models represent accumulated expert knowledge generated over years, which allows the simulation of system behavior in the face of perturbations (27). Dynamic simulations of process models offer a cost-effective approach for exploring design spaces before significant process development is carried out and can be used from the very earliest development stages through scale-up and optimization of operating conditions (80). Therefore, process models have been widely recognized as a supporting tool for monitoring, control, and optimization of biochemical processes within the QbD paradigm.

Generally, a process model consists of a mathematical representation of the interrelationships between process parameters and process outputs or quality attributes. Models can be developed to describe a variety of operations, ranging from upstream to downstream unit operations, in addition to integrated models that encompass series of operations (80). Process models have often been based on mechanistic knowledge about the underlying processes on a physical, chemical, or biological level. These models, also known as phenomenological models, not only have the advantage of encompassing a summary of available knowledge, but also provide value in planning experiments, or in determining which CPPs need to be monitored and controlled tightly (81,82). The required mechanistic knowledge for setting up models has been traditionally based on experimental observations leading to formulation of hypotheses on a case-by-case basis. The formulation of a well-regarded mechanistic model of overflow metabolism in biological organisms serves as an example (83). There is a clear trend towards development of methods which aid the identification and testing of various hypotheses in a fast and reliable manner, so that the time and cost required for setting up a robust mechanistic model for an unknown system is reduced (84). In our review of the available literature, we identified a gap with respect to KM strategies that support this aim: supporting mechanistic model development by summarizing existing process knowledge in an ordered manner.

Conversely, data-driven models rely on statistical inferences based on training datasets. While advantageous in cases where mechanistic process knowledge is scarce; the need for extensive training datasets is often a limiting factor for the applicability of such models during initial process development stages. While useful for data mining, hypothesis generation, and analysis of multivariate datasets generated by PAT instrumentations (22,85), the parameters of statistical models are more difficult to interpret; therefore, are not as useful as mechanistic models to serve as a store of available process knowledge.

In the future, the use of mechanistic models as efficient knowledge management and storage tools could be enhanced by combination with other technologies, such as ontologies. Since, semantic technologies allow for the standardization of a system’s knowledge content and the readability by machines, automated generation of mechanistic models based on collection of hypotheses from multiple actors can be envisioned. Ontological-modeling approaches, such as OntoMODEL, have been previously developed (19). This example represents an ontological tool for management of mathematical models, model storage, usage, and simulation studies. These ideas are also explored in the development of applied decision support systems for pharmaceutical manufacture (86), as well to mathematical knowledge modeling to define the processing route for manufacturing through a guideline’s execution system (87, 88). Currently, there is an opportunity to further explore the possibilities offered by the combination of ontologies and classical modelling approaches, as they could play an important role for knowledge transfer using an elegant and effective method for information codification.

## CONCLUSIONS

Knowledge must be analyzed according to the principles of quality risk management, prioritizing valuable information in data-rich environments. This constitutes the foundation of the QbD philosophy, where knowledge and risk management become the main enablers of a lifecycle approach. RM serves as a useful approach both for aggregation of information that is required to perform key QbD related activities such as design space definition as well as for managing information during the lifecycle of a commercial product. However, while RM has been explored in detail due to higher attention by the regulatory agencies, KM has received relatively scant attention. As the pharmaceutical industry grows in complexity, KM has gained a prominent seat, enhancing and being enhanced by the QbD initiative as a core technology in a true pharmaceutical quality system.

A simple yet effective classification of knowledge sources can be achieved based on their nature and criticality, reflecting the major sources of knowledge being addressed, namely information stemming from development activities, manufacturing experience, and knowledge from technology transfer activities. Further, KM tools can be catalogued with respect with the main function they perform: acquisition, analysis, storage and dissemination. These tools are supported by knowledge indicators, strategies, models and frameworks.

In our review of existing studies on the topic of KM related to QbD, we highlight that knowledge from transfer activities is not sufficiently covered by the current KM tools. However, there is a predominant use of statistical analyzers for the management of knowledge from manufacturing activities. On the other hand, databases, visualization tools, retrieval and data mining tools are focused on capture of prior knowledge. Collaboration and document management tools are mainly linked to knowledge from development studies. There are some tools that are applied holistically: taxonomies and ontologies, intelligent agents, network and web technologies, and models and simulation. However, taxonomies and intelligent agents which are highly quoted in literature and relevant in accelerating bioprocess development and commercialization, are conversely perceived as less important by the industrial counterparts. We found major gaps that could help to consolidate a true KM-based pharmaceutical quality systems if sufficiently developed: knowledge indicators, ontologies, and a combination of ontologies and mechanistic modeling approaches.

## Electronic supplementary material

ESM 1 (PDF 165 kb)

## ABBREVIATIONS

CPP: Critical process parameters
CQA: Critical quality attributes
ICH: International conference on harmonisation of technical requirements for registration of pharmaceuticals for human use
KM: Knowledge management
PAT: Process analytical technologies
QbD: Quality by design
RM: Risk management
