# Supplementary material for: Role of Knowledge Management in Development and Lifecycle Management of Biopharmaceuticals
Source: Pharm Res. 2016 Oct 26;34(2):243–56. doi: 10.1007/s11095-016-2043-9 (PMC5236082; doi:10.1007/s11095-016-2043-9)
Supplement: Supplementary file 1 — (PDF 165 kb) [file 11095_2016_2043_MOESM1_ESM.pdf]

Systematic review protocol

Role of Knowledge Management in Development and Lifecycle Management of Biopharmaceuticals

Primary reviewer: Oscar Fabián García Aponte

Secondary reviewer: Aydin Golabgir Anbarani

Reviewing process start date: October 16 2014

Institution conducting the review: Bioprocess Technology, Vienna University of Technology

1. Objective

The objective of this review is to analyze the records found in some databases, according to the frequency of the knowledge sources managed, pharmaceutical applications pursuit and Knowledge Management (KM) tools used in the context of the ICH Q8 to Q10 guidelines, gaining insights about the status of various KM tools within the frame of the Quality by Design (QbD) philosophy. Therefore, this study will address the following questions:

- Is there a homogeneous distribution of frequency among the different KM tools applied in the QbD context?
- Are the Knowledge sources adequately covered by the tools used?
- Which are the most frequent objectives pursuit by the application of the KM tools to QbD?
- How are the knowledge sources and the QbD objectives connected through the KM tools?

2. Inclusion criteria

Inclusion criteria will be used for determination of the suitability and the relevance of studies for this review. The criteria will be utilized on two specific occasions:

a) Inclusion criteria will be used to judge the suitability and pertinence of studies for retrieval from the database list searches, assessing the titles and, if needed, the abstracts of the evaluated papers. Included studies in this selection have to reach all the following criteria:

1. *Language:* English, German, Spanish, French and Portuguese.
2. *Paper status:* At least approved for publication.
3. *Title:* Must be clearly the title of a research or review paper, excluding codes, single worded titles and unintelligible characters combinations.

b) Inclusion criteria will be employed when the complete articles, selected from the previous stage, had been retrieved to determine if they should be used for data extraction in the review. This study will include all types of research addressing KM in the scope covered by the ICH Q guidelines. Included studies in this selection have to reach all the following criteria:

4. *Productive sector*: Chemical, Pharmaceutical, Cosmetics, Biotechnological and Food research and/or manufacture.
5. *Core issues:* Data, information or knowledge management through the specific application of one or more tools
6. *Activities studied:* Research and development, manufacture, transfer, data or information management.

3. Exclusion criteria

Exclusion criteria will be used as a part of this review to aid in the screening process that decides which studies would not be accepted in this study. Some studies will be excluded if they are presented in the exclusion criteria which are:

- Articles published which use the same tool by the same authors in the same context and the same objective.
- Articles mentioning “knowledge” in their structure but with no relation with any kind of management.

4. Search strategy

A comprehensive and exhaustive search will be undertaken of electronic databases. Searching terms and searching steps will be used as the following below:

Searching terms: Searching terms will generated from the combination of two concepts: knowledge management and an application field. The first one will be always used, the second one will be used with the conditional “OR”, following this pattern:

“Knowledge Management” AND (“pharmaceutical” OR “drugs” OR “biotechnology” OR “chemical”)

In addition, searching for different forms of a word using truncation symbol and wildcard could be used to search for all words beginning with a particular based term and alternative spellings, in case that the previous search equation gives no results.

If the database allows it, this search equation will be limited to Title, Abstract and Keywords search.

Searching steps:

The searching process will be simple and will not include further evaluation of journals found interesting for the topic of research or further analysis of the references found in the primary papers selected.

1. A comprehensive search of each database using all optimal search terms, as stated previously, will be undertaken.
2. To keep up to date, the specific database will be searched monthly to recognise published studies during conducting this review.

The electronic databases to be searched are:

- Scopus
- Science Direct
- Embase
- IEEE
- Springer
- Taylor & Francis

5. Quality assessment

Qualitative and quantitative papers selected for retrieval will be assessed by two independent reviewers for methodological validity prior to inclusion in the review, using criteria agreed between them and based on a review of methodological literature from the fields of knowledge management. Any disagreements that arise between the reviewers will be resolved through discussion, or with a third reviewer.

The robustness of the publication will be judged on the basis of whether the study provides sufficiently detailed outcome measures to allow conclusions to be made about the impact of the KM tool used on the development of the activities defined in the scope of this review.

Finally, the following topics will be evaluated always in those papers selected for a full text analysis, excluding them if they cannot meet an adequate level of quality.

1. Theoretical approach
   - Is a qualitative approach appropriate? Or is a quantitative approach appropriate?
   - Is the study clear in what it seeks to do?
2. Study Design
   - How defensible/rigorous is the research design/methodology?
3. Trustworthiness
   - Is the context clearly described?
   - Were the methods reliable?
4. Analysis
   - Is the data analysis sufficiently rigorous?
   - Are the findings convincing?
   - Adequacy of Conclusions
5. Ethics
   - How clear and coherent is the reporting of ethics?

6. Data collection

A data extraction sheet (see appendix) will be utilized for collecting data and minimise the risk of error during the transcription of data. Studies that are identified as relevant in accordance with the inclusion criteria will be obtained and classified with a data extraction sheet related to the objective of this review, which are:

- Year of publication
- Title of the study
- Source of knowledge managed
- Specific KM tool used
- General KM tool group
- Specific objective pursuit

END OF PROTOCOL
